# A histological evaluation of the surgical margins from human oral fibrous-epithelial lesions excised with CO2 laser, Diode laser, Er:YAG laser, Nd:YAG laser, electrosurgical scalpel and cold scalpel

**DOI:** 10.4317/medoral.22819

**Published:** 2019-03

**Authors:** Luís Monteiro, Maria-Leonor Delgado, Fernanda Garcês, Mariana Machado, Fernando Ferreira, Marco Martins, Filomena Salazar, José-Júlio Pacheco

**Affiliations:** 1 Medicine and Oral Surgery Department and Cancer Research Group - IINFACTS, University Institute of Health Sciences, CESPU, Paredes 4585-116, Portugal; 2 Pathology Department and Cancer Research Group - IINFACTS, University Institute of Health Sciences, CESPU, Paredes 4585-116, Portugal; 3 Physiology Department, University Institute of Health Sciences, Paredes 4585-116, Portugal; 4 Medicine and Oral Surgery Department and Oral Diseases Group - IINFACTS, University Institute of Health Sciences, CESPU, Paredes 4585-116, Portugal

## Abstract

**Background:**

We aim to evaluate the presence of histological artefacts in the surgical margins of human oral fibro-epithelial hyperplasias excised with lasers of different wavelengths, and also electrosurgical scalpel and cold scalpel. Moreover, we aim to determine if some of these instruments could impair the normal histological diagnosis of these lesions.

**Material and Methods:**

We included 130 consecutive surgical samples of 80 females and 50 males (mean age of 53.82±16.55) with a histological diagnosis of an oral benign fibrous-epithelial hyperplasias. The samples were categorized into 6 groups according to the type of instrument used: CO2 laser group, diode laser group, Er:YAG laser group, Nd:YAG laser group, electrosurgical scalpel group and cold scalpel group. Histological instrument-induced changes were microscopic evaluated and related with clinical and pathological variables.

**Results:**

The instrument with highest tissue damage extension (TDE) was the electrosurgical scalpel (1002.2µm±434.92), followed by diode laser (913.73 µm±322.45), Nd:YAG (899.83µm±327.75), CO2 laser (538.37µm±170.50), Er:YAG laser (166.47µm±123.85), and at last with fewer alterations the cold scalpel group (2.36µm±7.27) (*P*< 0.001). The most regular incision was observed in CO2 laser group, followed by Er:YAG laser, Nd:YAG laser, electrosurgical scalpel and diode laser group with the less regular incision using cold scalpel as comparison (*P*< 0.001). A correlation was found between the incision score and TDE (*P*< 0.001). Regarding histological diagnosis, no case showed any limitation of diagnosis related with the use of any instrument evaluated.

**Conclusions:**

Our results suggest that lasers can be used for the excision of oral benign fibrous-epithelial hyperplasias, without hispathological diagnosis limitations, as long as the physical properties of each laser are known and respected. Er:YAG laser have shown to be a laser with few tissue damage extension and with good incision regularity, been a possible instrument of choice for the surgical removal of these lesions.

** Key words:**CO2 laser, diode laser, Er:YAG laser, Nd:YAG laser, oral mucosa.

## Introduction

Oral cavity includes a unique combination of several tissues that contributes to the existence of many physiological functions such fonation, chewing or breathing. However, in the presence of chronic fricctional stimuli, oral tissues can produce some pathological adaptations resulting in oral lesions such as fibroepithelial polyps or other fibroepithelial growths. These benign fibroepithelial hyperplastic lesions correspond to the most common lesions of the oral mucosa (1) and their treatment includes complete excision. Several surgical options have been suggested including excision with cold scalpel, electrosurgical scalpel or with lasers (1,2).

In the last decades laser usefulness have been reported in many oral surgical procedures with several advantages over classical surgical methods. These advantages include the haemostatic capacity enabling a bloodless surgical field, the decontamination properties, significant decrease in postoperative pain, inflammation and infection, and second intention healing without need for sutures (2-6).

Different type of lasers have been used to treat benign fibro-epithelial hyperplastic lesions such as carbon dioxide (CO2) laser (10600nm), neodymium-yttrium-aluminum-garnet (Nd:YAG) laser (1064nm), diode laser (800-980nm), potassium-titanium-phosphate (KTP) laser (532nm), and erbium-yttrium-aluminum-garnet (Er:YAG) laser (2940nm) (7-12).

During laser irradiation, the photon energy is transformed into thermic energy in oral tissues where absortion occurs producing thermal changes that can range from temporary heating (42º to 50º), to protein denaturation and coagulation (60º), vaporization and ablation (100º), or even to carbonization (at temperatures above 200º). This photothermal effect can cause microscopic artifacts in the peri-incisional area of the lesion (8). Reports of epithelial and connective tissue artefacts caused by lasers have been reported including nuclear changes such as hyperchromic nuclei, intracellular vacuolization, cell fusion, loss of cell attachment, carbonization and desiccation (8,10,13,14). They could lead to imprecise histological observations such as the existence of pseudodysplasic changes that could impair or at least interfere with histopathological diagnosis of oral soft tissue lesions (15-17).

The aim of this study was to evaluate the microscopic morphological changes in the surgical margins of human oral fibro-epithelial hyperplasias excised with CO2, diode, Nd:YAG or Er:YAG lasers, electrosurgical scalpel and cold scalpel. We aimed also to determine which instrument produce highest and lower tissue damage and finally if some of these instruments could impair the normal histological diagnosis of these lesions.

## Material and Methods

-Study population

We retrospectively included patients with diagnosis of benign fibro-epithelial oral lesions located on oral cavity with indication to excision between January 2010 to July of 2018 in the Oral Laser Unit of the Nova Saúde SA – University Institute of Health Sciences, Oporto, Portugal. The study was undertaken under the permission of the institutional ethical board of the University (IUCS Ethical Council) and performed in accordance with the Declaration of Helsinki. All patients provided written informed consent for the surgical treatment and for the use of patient information’s data. Detailed data retrieved included several demographic, clinical, and pathological variables such as patient’s age, gender, lesion location, size of the lesion, type of surgical procedure, histological diagnosis, and follow-up information. Patients were included if they had 18 or more years-old, a histological diagnosis of a benign epithelial, fibrous or fibrous-epithelial hyperplasia, and had a lesion within oral cavity (ICD, C01-06). Patients with systemic diseases (e.g. non-controlled diabetes mellitus, haemorragic diathesis, infectious diseases) or with oral anticoagulant or immunosuppressant medication were excluded. From initially search of cases we find 142 patients within the period of the study, but 12 were exclude because incomplete clinical data or because the histological specimen was not present for histological analysis.

The cases were divided into 6 groups depending of the type of instrument used for the excision procedure: Group 1 (CO2 laser); group 2 (diode laser), group 3 (Er:YAG laser); group 4 (Nd:YAG laser); group 5 (electrosurgical scalpel); and group 6 (cold scalpel). The use of a particular instrument to another had a random selection.

Final sample was composed by 130 patients, 80 females and 50 males, with a mean age of 53.82±16.55 (range 18 to 85 year-old). The lesions were located on buccal-vestibular-lip mucosae (n=70), in gingivae mucosae (n=32), in the hard palate (n=7), in the soft palate (n=4), and in tongue (n=17). Grouping locations based on histological origin 91 cases were located on non-keratinized mucosa and 39 on keratinized mucosa ( Table 1). Regarding histopathological diagnosis all lesions included as benign epithelial and fibrous overgrowths corresponded to fibro-epithelial hyperplasias, denture-related fibrous hyperplasias, fibromas, and fibropapillomas.

**Table 1:**
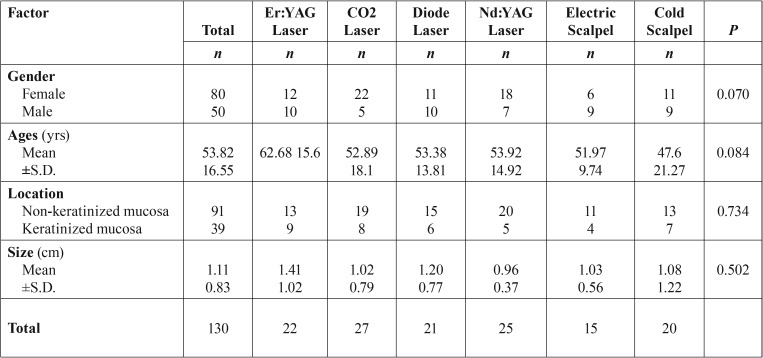
Analysis of the patients characteristics by instrument group.

All excision procedures were performed by the same operator (LM) using local anaesthesia with lidocaine with vasoconstrictor 2% XILONIBSA®.

The characteristics and specifications of the instruments (following indications of each device manufacture for oral mucosa lesion excision) were: CO2 laser (wavelength of 10600nm - DEKA® Smart US 20D, Firenze, Italy) was used with an angulated mirror (120º) handpiece, focalized mode with 0.5-mm spot, with a frequency of 80Hz, an energy of 50mJ and power of 4W (power density of 2040.8W/cm2 and fluence of 40.8 J/cm2); diode laser (wavelength of 980nm - LITEMEDICS®, Brendola, Italy) was used with a fiber of 300-µm in contact mode, with a frequency of 50Hz, an energy of 70mJ and a power of 3.5W (power density of 4957.5W/cm2 and fluence of 99.2 J/cm2); Nd:YAG laser (wavelength of 1064nm Smarty-A10 DEKA® Smart US 20D, Firenze, Italy) was used with a fiber of 300-µm in contact mode, with frequency of 40Hz, “short pulse”, energy of 100mJ and power of 4W (Power Density: 5665.7 W/cm2; Fluence: 141.6 J/cm2); Er:YAG laser (wavelength of 2940nm; FOTONA®, LigthWalker, Slovenia) was used with a angulated mirror (90º) handpiece (H02), focalized with a spot of 0.5-mm, with frequency of 20Hz, “Long pulse” (LP), energy of 200mJ and power of 4W (Power Density: 2040.8W/cm2; Fluence: 102 J/cm2); electrosurgical scalpel (Electrosurgical Knife - CARLO DI GIORGI®, Milan, Italy) was used with a thin straight electrode (diameter: 0.22-mm), in contact mode, in cutting/coagulation mode, at 5W of power; and traditional excision with cold scalpel was performed with a KIATO® scalpel blade number 15C with a number 3 handle. Usual safety precautions related with each instrument for protecting the operator, patient, and assistant were followed.

Except for scalpel surgeries, in the all other cases the wound surgery was left open to promote granulation and secondary epithelialization. Postoperatively, paracetamol 1g (SOS) was prescribed to all patients. All surgical specimens were immediately fixed in a 10% buffered formalin solution and were send for histopathological evaluation.

-Histologic evaluation

Serial sections were performed with 3µm thickness and stained with haematoxylin-eosin (HE) for histopathological diagnosis and tissue damage extent (TDE) analysis. All histological sections were evaluated using a ZEISS AxioLab A1® microscope (Carl Zeiss Microscopy GmbH, Jena, Germany), with a ZEISS Axiocam 105 color® and ZEISS Zen2® software, and performed by an experienced pathologist, blinded to the type of surgical instrument used.

The presence of histological alterations adjacent to the surgical margins were evaluated (at magnification of 40x) according the criteria established by Vescovi *et al.* (8). This consisted in: epithelial changes including nuclear changes (picnotic, spindle-like and hyperchromic nuclei), cytoplasm changes (hyperchromic cytoplasm, cell fusion, and loss of cell attachment), and possible loss of intraepithelial and subepithelial adhesion; modification of connective tissue including carbonization and desiccation; presence or absence of vascular alterations (presence of thrombosed or collapsed blood or lymphatic vessels); morphology and regularity of the incision on a scale of 0 to 4 where level 4 represents the highest quality and 0 the worst incisional quality and finally a tissue damage extension (TDE) (expressed in microns) was measured in the most evident damage area in the surgical margins, from the greatest distance from the edge of the incision to the end of the laser thermal damage, perpendicular to the surgical margin, in the epithelial area and also in the connective area.

The mean size of specimen was 1.1±0.83cm (minimum - 0.4cm and maximum - 6cm). For statistical analysis the samples were categorized size into to the categories <1.1cm and ≥1.1cm ( Table 1).

-Statistical analysis

The data analysis was obtained by descriptive and inferential statistics, using the SPSS-24.0 software (Statistical Package for Social Sciences). The results were expressed in absolute and relative frequencies. We use firstly Shapiro-Wilk test to analyse the normality of the numerical variables in study, which showed a non-parametric distribution. In the view of this, non-parametric tests were used to analyze possible relations between continuous variables (Spearman correlation test, Mann-Whitney test, Kruskal-Wallis test) and categorical variables (Chi-Square test). Post hoc analyses were also used. Differences were considered statistically significant at *P*<0.05.

## Results

**Figure 1:**
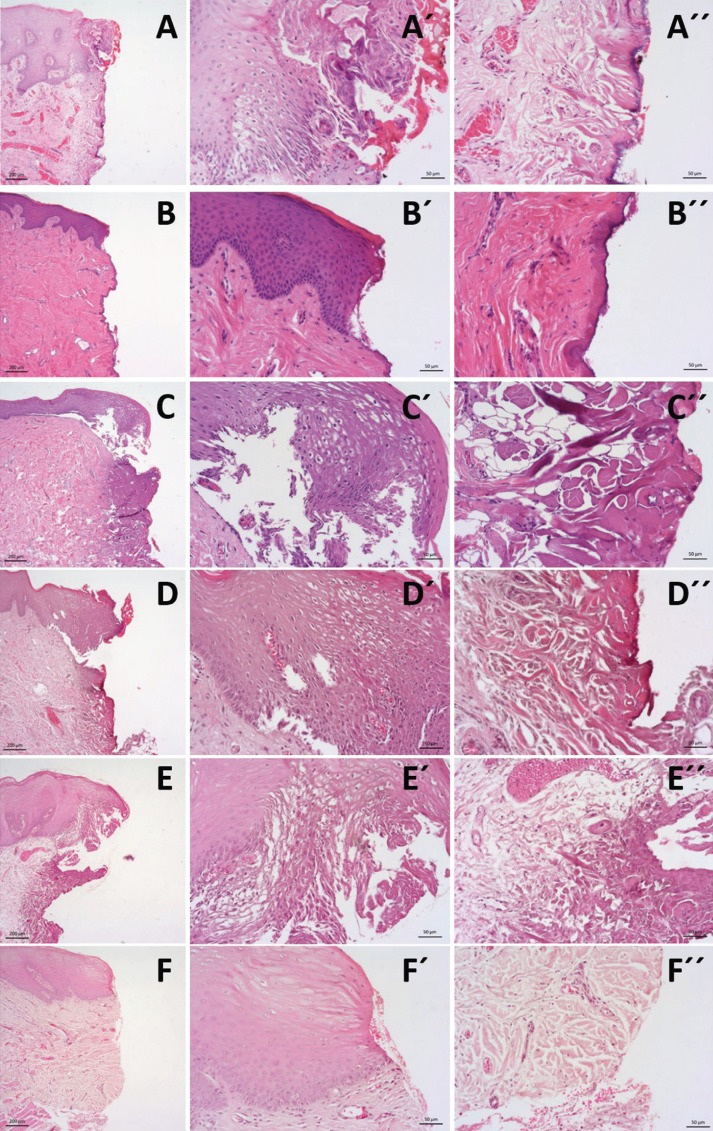
Histological images (haematoxylin and eosin staining) at magnification of 5x and 20x for epithelium view (´) and connective view (´´) of the surgical margins of tissue samples submitted to excision by the 6 groups of instruments: A –CO2 Laser; B –Er:YAG Laser; C –Diode Laser; D – Nd:YAG laser; E – electrical surgical scalpel; F – cold scalpel.

The presence of thermal artefacts according to the surgical instrument used are shown in  Table 2. On epithelial analysis, a significant difference between the groups was observed regarding the presence of nuclei alterations (*P* < 0.001), cytoplasm alterations (*P* < 0.001) and the presence of the loss of epithelial attachment (*P* < 0.001) ( Table 1). Regarding connective alterations, a significant difference was also observed between the instrument groups regarding the presence of carbonization (*P* < 0.001), desiccation (*P* < 0.001) and the presence vascular alterations (*P* < 0.001) ( Table 2) (Fig. 1). With these variables, we performed epithelial and connective scores with the sum of previous variables and find also significant differences for epithelial and connective scores (both *P* < 0.001). On post hoc analysis of epithelial score the significant differences were obtained between cold blade vs Er:YAG laser (*P* < 0.001), cold blade vs CO2 laser (*P* < 0.001), cold blade vs Nd:YAG laser (*P* < 0.001), cold blade vs diode laser (*P* < 0.001), cold blade vs electrosurgical scalpel (*P* < 0.001), Er:YAG laser vs Nd:YAG laser (*P* < 0.001), Er:YAG laser vs diode laser *P* < 0.001), and Er:YAG laser vs electrosurgical scalpel (*P* = 0.012). For connective score, significant differences were observed between cold blade vs Er:YAG laser (*P* < 0.001), cold blade vs CO2 laser (*P* < 0.001), cold blade vs Nd:YAG laser (*P* < 0.001), cold blade vs diode laser (*P* < 0.001), and cold blade vs electrosurgical scalpel (*P* < 0.001) ( Table 2).

**Table 2:**
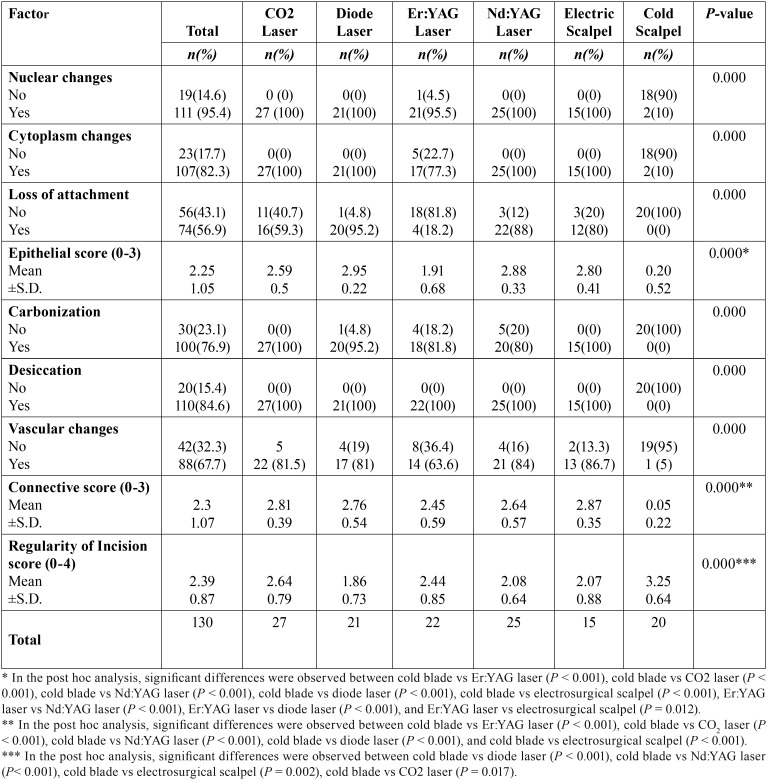
Epithelial and connective alterations by instrument group.

We also analysed the regularity of the incision made with each type of instrument ( Table 2). In overall, 56(43.1%) cases showed a regular incision. The most regular incision was obtained with the scalpel followed by CO2 laser, by Er:YAG laser, Nd:YAG laser, electrosurgical scalpel group and diode laser with the less regular incision (*P* < 0.001). In post hoc analysis of incision score the significant differences were obtained between cold blade vs diode laser (*P* < 0.001), cold blade vs Nd:YAG laser (*P* < 0.001), cold blade vs electrosurgical scalpel (*P* = 0.002), cold blade vs CO2 laser (*P* = 0.017), but not for the other remaining combinations (*P*>0.05).

**Figure 2:**
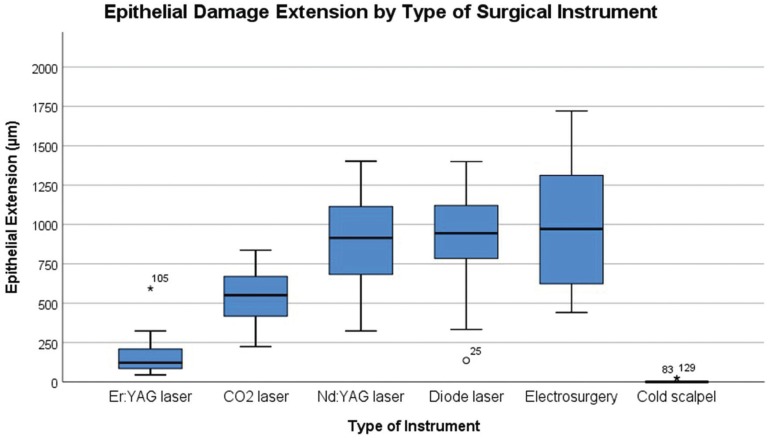
Box-plot of Epithelial Damage Extension (µm) according to Surgical Instruments.

**Figure 3:**
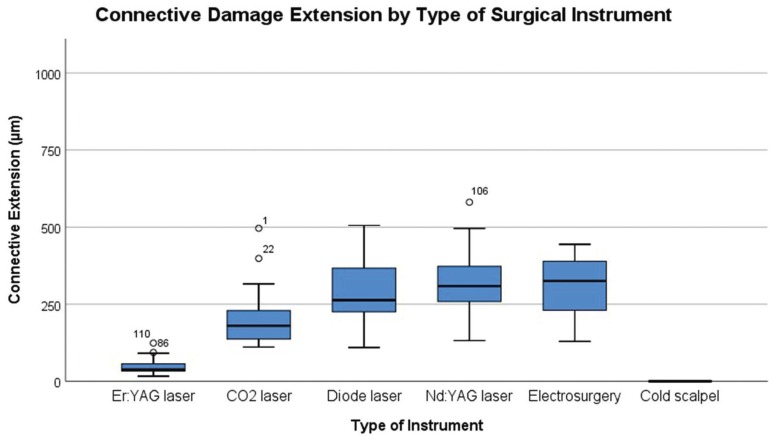
Box-plot of Connective Damage Extension (µm) according to Surgical Instruments.

The tissue damage extension (TDE) was evaluated in surgical margins of the samples at both epithelial and connective tissue level and the results are presented in  Table 3. The instrument with highest TDE in the epithelial zone was the electrosurgical scalpel (1002.2µm±434.92), followed by diode laser (913.73 µm±322.45), Nd:YAG (899.83µm±327.75), CO2 laser (538.37µm±170.50), Er:YAG laser (166.47µm±123.85), and at last with fewer alterations the scalpel group (2.36µm±7.27) (Fig. 2). In the connective zone, the highest TDE was observed in the electrosurgical scalpel group (393.80µm±359.11), followed by Nd:YAG (310.85µm±107.45), diode laser (284.81µm±110.56), CO2 laser (201.69µm±89.86), Er:YAG laser (48.54µm±26.09), and not detected in the scalpel group (0µm±0) (Fig. 3). These differences within all groups were significant in the epithelial zone (*P* < 0.001) and connective zone (*P* < 0.001). In particu-lar for epithelial zone, post hoc analysis showed significant differences between cold blade vs CO2 laser (*P* < 0.001), cold blade vs Nd:YAG laser (*P* < 0.001), cold blade vs diode laser (*P* < 0.001), cold blade vs electrosurgical scalpel (*P* < 0.001), and also between Er:YAG laser vs Nd:YAG laser (*P* < 0.001), Er:YAG laser vs diode laser (*P* < 0.001), Er:YAG laser vs electrosurgical scalpel (*P* < 0.001). Interestingly, significant differences were not found for cold blade vs Er:YAG laser (*P* > 0.05) and for the other remaining combinations (all with *P* > 0.05). In the post hoc analysis of connective zone, we observed significant differences between cold blade vs CO2 laser (*P* < 0.001), cold blade vs Nd:YAG laser (*P* < 0.001), cold blade vs diode laser (*P* < 0.001), cold blade vs electrosurgical scalpel (*P* < 0.001), and also between Er:YAG laser vs CO2 laser (*P* = 0.012), Er:YAG laser vs Nd:YAG laser (*P* < 0.001), Er:YAG laser vs diode laser (*P* < 0.001), Er:YAG laser vs electrosurgical scalpel (*P* < 0.001). But, interestingly, not for cold blade vs Er:YAG laser (*P* > 0.05) and for the other remaining combinations (all with P > 0.05).

**Table 3:**
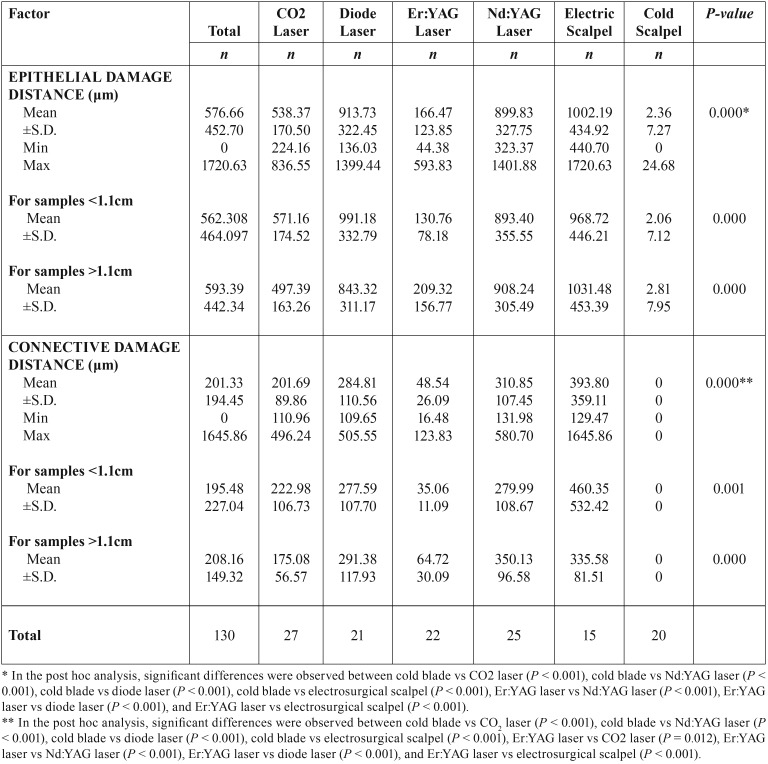
Tissue Thermal Damage Extension by by instrument group and size of specimen.

The TDE was higher in the epithelial part than in the connective part in all instruments but a significant correlation was observed between them (r = 0.843; *P* < 0.001). Generally, it was observed a higher TDE in the presence of a greater number of changes within nucleus (*P* < 0.001), cytoplasm (*P* < 0.001), and loss of adherence (*P* < 0.001) or carbonization (*P* < 0.001), desiccation (*P* < 0.001) or vascular changes (*P* < 0.001).

We didn´t find any correlation between the size of the lesions and epithelial TDE (*P* = 0.661) or connective TDE (*P* = 0.288) and neither with nuclear, cytoplasm, loss of attachment, carbonization, desiccation or vascular changes. We find a correlation between the incision score and epithelial TDE (r = -0.438; *P* < 0.001) or connective TDE (r = -0.467; *P* < 0.001) where cases with lower tissue damage distances had better quality of incisions.

Regarding histological diagnosis, no case showed any limitation of diagnosis related with the use of laser.

## Discussion

We aimed to analyze the morphological characteristics of the oral tissues submitted to different kinds of surgical instruments specially lasers and electrosurgical scalpel and compare them with traditional scalpel. We observed the presence of cytological artifacts in the surgical margins in almost the laser instruments and electrosurgical scalpel groups. However, artifacts caused by different types of laser were limited to a small area of tissue and did not affect the entire fragment making possible the histological diagnosis without any referred limitation in the present sample.

We have evaluated several histological variables at the margins of the excised lesions, not only in the epithelial layer but also in the connective part. In the epithelium analysis, we observed some degree of nuclear and cytoplasmic alterations in almost every case of laser or electrosurgical scalpel instruments comparing with cold scalpel. Er:YAG laser had fewer cases with these alterations than the others specially cytoplasm artefacts as described by Suter *et al.* (12) in Human oral specimens and Merigo *et al.* (13) in an *ex vivo* study. As expected, cold scalpel was the instrument with fewer alterations but, interestingly, we found some cases with nuclear and cytoplasm alterations also in this group. This could be related with some inappropriate manipulation of the specimens by the operator or with technical processing.

Loss of cell or epithelial adherence were noted in some cases of laser or electrosurgical scalpel instrument’s groups. Higher number of images with loss of attachment were recorded in diode laser, contrasting with Er:YAG laser, the group with fewer adherence alterations after cold scalpel. We also constructed an epithelial score to evaluate the accumulate amount of artefacts on epithelium layer and in accordance with previous variables the Er:YAG laser was the instrument, after cold scalpel, with less degree of epithelium alterations score and diode laser the instrument with highest epithelium score.

In the connective analysis, all cases showed dissecation or hyalinization of connective margins contrasting with the absence of this alteration on cold scalpel group. The presence of carbonization was present in laser and electrosurgical scalpel groups although, Er:YAG laser group was the one with less number of this observation, similar to the reported by other authors (13). This was expected as all kind of surgical lasers produces a photothermal effect. Indeed, CO2 lasers and electrosurgical scalpel showed some degree of carbonization in all cases evaluated. Additionally, to the thermal effect, these two instruments have a superficial absorption/action which could result in a higher degree of carbonization in the most superficial part of the tissue.

Considering the vascular changes, we could observe that there is a clearly difference between eletrocautery/laser groups comparing with scalpel group. All laser instruments and electrosurgical scalpel presented a high level of vascular alterations with electrosurgical scalpel group with high number of thrombosed or collapsed vessels and Er:YAG the group with fewer vascular alterations. This results confirm the usefulness of electrical scalpel, Nd:YAG, CO2 and diode lasers, as instrument’s with good coagulation capacity and indicated to when the hemostasis is mandatory. Er: YAG laser is sometimes regarding in the literature as wavelenght not suitable for surgery in oral soft tissues because has no haemostatic properties (2). However, our results do not support this, as we found more than half of the patients submited to Er:YAG surgery had vascular alterations identified as thrombosed or collapsed vessels. This may be related to the non-use of water/air spray or the use of a long pulse duration in these cases which raises the temperature allowing a better coagulation. Taking in to account also the minimal artefactual damage caused by this laser, we think that this instrument should be regarded as an interesting and efficient option for surgical excision of oral soft lesions.

An important issue regarding laser application in surgery is the quality of a good and regular incision. We observed that the best incision score was obtained, as expected, in the scalpel group and followed by CO2 laser. However, we note not that all cases of scalpel group had a regular incision. Moreover we find also a good regularity of incision for Er:YAG laser. Indeed, in post hoc analysis we didn’t find significant differences between cold blade and CO2 laser or Er:YAG laser which could suggest that these two lasers have a regularity score very similar to a cold scalpel. This is in contrast with some authors that have reported a poor incision regularity for Er:YAG indicating the creation of micro explosions on the tissue as a cause for the irregularity of the incision (8,13). Nevertheless, the parameters that we used, as a high frequency, long pulse, absence of water/air spray or the new improvals in the technology of the Er:YAG lasers could led to a better incision quality compared with other parameters (8,13,18). Other possibility is the fact that the fewer artefactual alterations in margins could improve the regularity of the incision.

Regarding the histological analysis of the thermal damage distance in the surgical margins we observed that the distance of damage produced by the instruments were higher in the epithelial part than in the connective part and were correlated one which the other.

We also found that the presence of high level of histological/cellular changes were directly related with higher distance of thermal damage, as described previously (14).

The group with smaller distance of damage was the Er:YAG laser with a mean of 166.47µm±123.85μm. These results are in agreement with some studies in human specimens (12,19,20) and ex vivo studies (13-15,21) concluding that the thermal damages caused by this laser are minimal. By contrast, electrosurgical scalpel presented the highest extent damage with maximum value of 1720.63μm (mean of 1002.19µm±434.92) followed by diode and Nd:YAG laser. The thermal artefacts cause by electrosurgical scalpel are well known (22,23) and related with thermal properties of this kind of instrument. Regarding diode laser, Cercadillo-Ibarguren *et al.* (21), observed that diode group presented the most significant thermal cell damage, where large areas of carbonization and artifacts were observed. Diode and Nd:YAG have been reported as lasers with high thermal damage in the margins as reported by Romeo *et al.* (11) when testing the effect of different lasers and Vescovi et al. comparing the Nd:YAG laser with traditional scalpel (8). Merigo *et al.* (13) observed an increase in the temperature in depth in the diode and Nd:YAG lasers, and related with higher extent of tissue change. Diode lasers and Nd:YAG lasers have a deep absorption distance as the laser is better absorbed by hemoglobin and melanin by allowing a deep penetration of energy in the tissue which could be related with high distance of damage (8,13). It is interesting to note that in connective TDE evaluation Nd:YAG laser had a higher value than diode that could be explained by the fact that Nd:YAG has a more deeper action than diode laser.

Vescovi *et al.* (8) reported the presence of higher thermal damage distance in samples with reduced size (<7mm). Angiero *et al.* (10), also reported a relation of the presence of artefacts with size observing a limitation in the diagnosis in samples <3mm. We could not find this relation of size of the sample and the presence of artefacts in surgical margins in our samples, however all our samples size were superior to 4mm.

No limitations on histopathological reports was found in the present sample. This is an important result indicating that these instruments could be used for an efficient surgical intervention and with the possibility of a pathological report without significant histological limitations. This has been confirmed by other authors (8,11,12) in human samples and in *ex vivo* studies (24-26). Interesting none of the lasers cause damaged in surgical margins superior to 1.5mm. In the view of this, the operator should have this in mind on the planning of surgical procedure and include an additional millimeter (1-2mm depending of laser wavelength) at healthy tissue to minimize this possible damage. Moreover, an experienced practice, education and training in the areas of laser are important to get the best results with these instruments.

We acknowledge some limitations in our study. We have included different locations among oral cavity. Nevertheless, we didn’t found differences on sample constitution of cases located on non-keratinized mucosa or keratinized mucosa. As there are no stablished protocols to determine the parameters of use of each laser, we used the recommended parameters by the manufacture for each instrument for this type of surgery and tissue. Nevertheless, we included a significant number of patients in each group in a randomized manner with a control group and also specimens were histopathological evaluated blinded to the characteristics and method used to the excision, although performed by one (but experienced) pathologist. To our knowledge this is the first study of excisions of human oral fibro-epithelial hyperplasias with different groups of instruments including the most used wavelength lasers in Dentistry such as CO2, diode, Nd:YAG or Er:YAG lasers, electrosurgical scalpel, and also a control cold scalpel group.

As conclusion, our results showed that Er:YAG laser is the laser with lowest capacity for causing tissue damage in the surgical margins and electrosurgical scalpel the instrument with highest tissue damage extension. However, none of the groups were related with any limitation regarding histopathological diagnosis which means that when these instruments are correctly used and with the awareness of their own properties they can be a safe and efficient instrument’s for the excision of human oral fibro-epithelial hyperplasia’s.
